# The ingenious drainage system controls persistent duodenal stump fistula due to pancreatic fistula after subtotal gastrectomy for advanced gastric cancer

**DOI:** 10.1093/jscr/rjae444

**Published:** 2024-07-03

**Authors:** Kazuya Moriwake, Hiroshi Isozaki, Takehiro Takama, Shigeki Murakami, Sasau Matsumoto

**Affiliations:** Department of Surgery, Oomoto Hospital, 1-1-5 Oomoto, Kita-ku, Okayama 700-0924, Japan; Department of Surgery, Oomoto Hospital, 1-1-5 Oomoto, Kita-ku, Okayama 700-0924, Japan; Department of Surgery, Oomoto Hospital, 1-1-5 Oomoto, Kita-ku, Okayama 700-0924, Japan; Department of Surgery, Oomoto Hospital, 1-1-5 Oomoto, Kita-ku, Okayama 700-0924, Japan; Department of Surgery, Oomoto Hospital, 1-1-5 Oomoto, Kita-ku, Okayama 700-0924, Japan

**Keywords:** complication, duodenal stump fistula, gastrectomy, gastric cancer, multiple drainage system, pancreatic fistula

## Abstract

Duodenal stump fistula (DSF) is a dangerous complication after gastrectomy. There is no consensus on the management of DSF. Sometimes, emergency surgery may be necessary. We present the case who underwent subtotal gastrectomy with Roux-en-Y reconstruction for advanced gastric cancer. After that surgery, we diagnosed DSF due to pancreatic fistula, and performed reoperation because of hemodynamic instability due to diffuse peritonitis and sepsis. We resected the stump and closed with handsewn suturing and inserted three intra-abdominal drainage tubes, including a dual drainage tube around the duodenal stump. Although there was a recurrence of DSF, because of the continuous and absolute drainage, the patient improved and discharged on postoperative Day 59. From this experience, diligent debridement and a continuous suction dual drainage system, intraluminal drain of the duodenum, and biliary diversion may be an effective surgical management for DFS.

## Introduction

Duodenal stump fistula (DSF) is a lethal complication of gastrectomy. Although its incidence rate is low, mortality rate is high. Several managements for DSF have been reported, but if the patient becomes hemodynamically unstable, emergent operation is required. Herein, we report an ingenious surgical drainage system for persistent DSF.

## Case report

**Figure 1:**
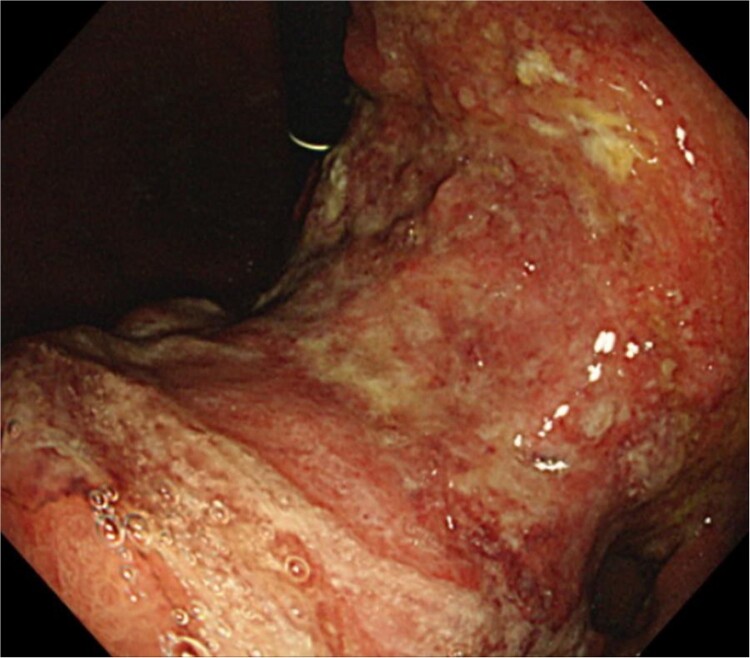
Clinical findings of endoscopy. Upper endoscopy showing a large tumor from body to antrum of the stomach.

**Figure 2:**
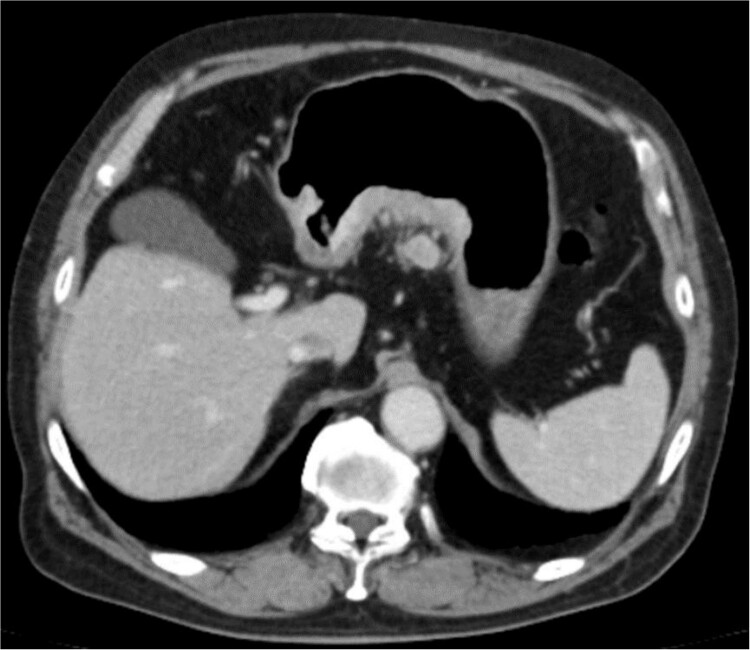
Clinical findings of contrast-enhanced CT. CT revealed the thickness of gastric wall and enlarged lymph nodes of lesser curvature side.

An 83-year-old man visited due to discomfort in the stomach. The patient underwent upper endoscopy that revealed a large tumor in the stomach (Fig. 1). The histological diagnosis was moderately differentiated tubular adenocarcinoma. Computed tomography (CT) revealed thickening of the gastric wall and bulky lymph nodes around the pancreas (Fig. 2). The patient underwent subtotal gastrectomy with Roux-en-Y reconstruction. The metastatic lymph nodes around the pancreas were removed. Abdominal drainage from Winslow’s foramen was performed. The pathological result was pT4aN3aM0 and Stage IIIB [1].

**Figure 3:**
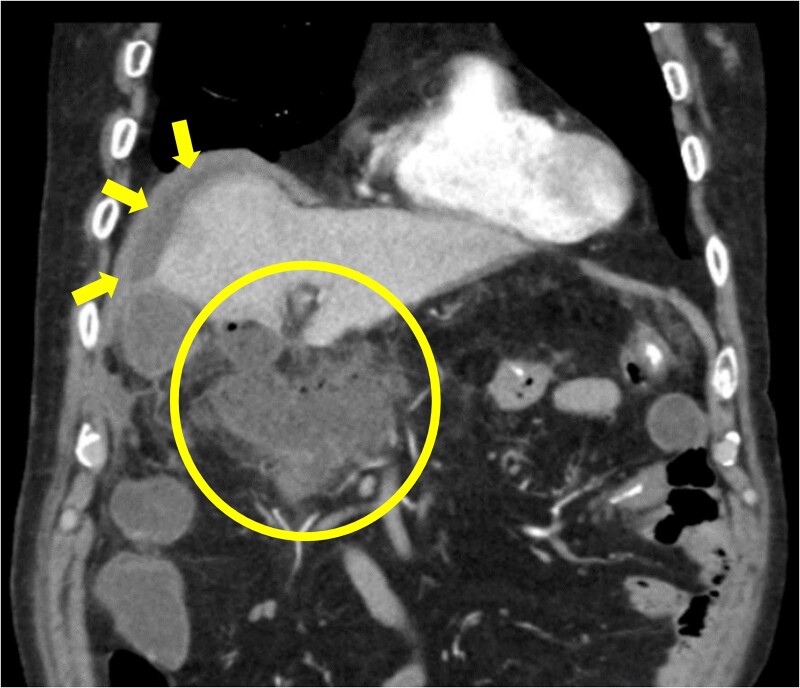
Diagnosis of postoperative duodenal perforation. CT showed ascites in subdiaphragmatic space (arrow) and around duodenal stump (circle) at POD 8.

**Figure 4:**
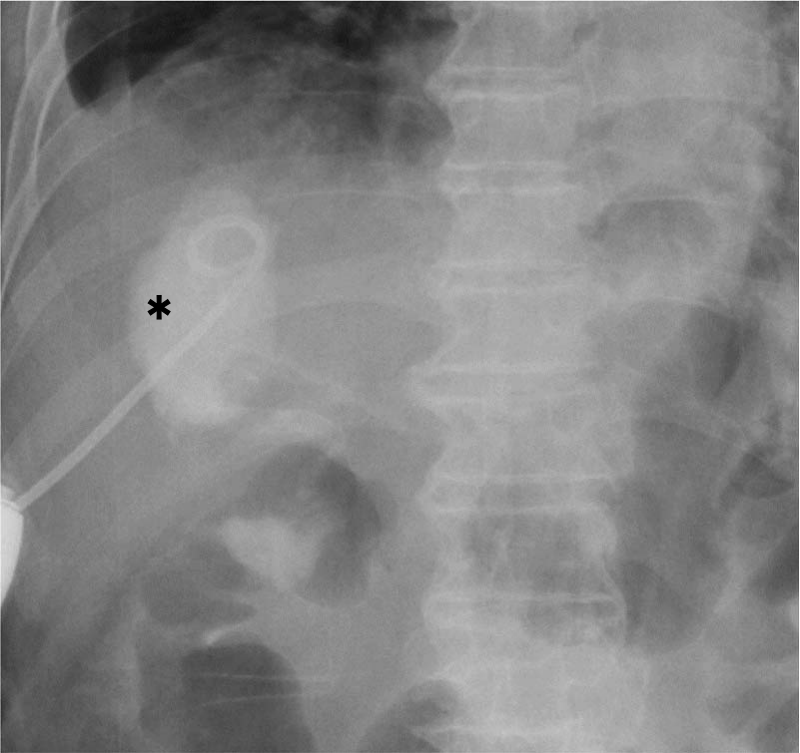
Primary drainage for duodenal fistula. As percutaneous approach, we inserted a pig-tail drainage tube to subdiaphragmatic space (asterisk) using X-ray fluoroscopy.

**Figure 5:**
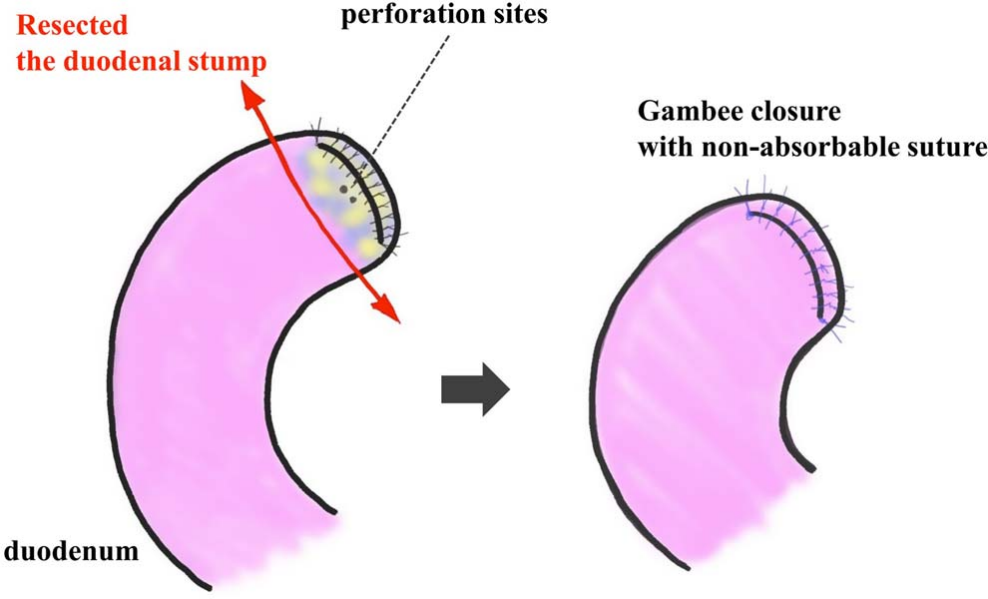
Resection and closure for two fistulas of the duodenum. Two pinhole perforation sites of the duodenum at the side of the anterior wall near the stump were identified. We resected the vulnerable duodenal stump, including the perforation site, by surgical scissor and close it by the interrupted single-layer suture (Gambee’s method) with unabsorbable 4–0 proline.

**Figure 6:**
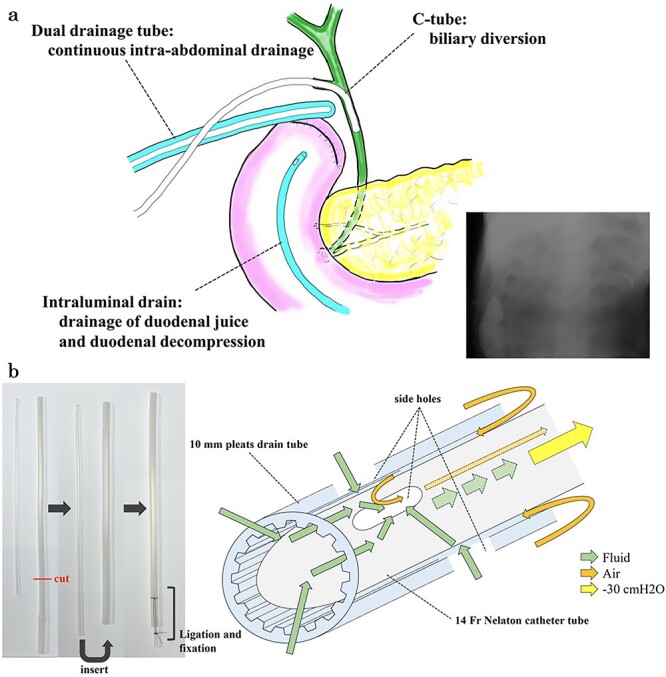
Multiple drainage system for the DSF and dual tube for continuous intra-abdominal suctional drainage and procedure of making. (a) In the reoperation, we placed three drainage systems for DSF: a C-tube from the cystic duct into the common bile duct, a dual drainage tube around the duodenal stump with continuous suction, and an intraluminal drain via the duodenum near the stump. (b) The dual drainage tube was made of 10 mm pleats and a 14 Fr Nelaton catheter tube. First, the tail of 10 mm pleats drain tube was cut. Secondly, 14 Fr Nelathon catheter tube was inserted into 10 mm pleats drain tube. At last, the tail of each tube was secured with ligation using 2–0 silk thread. Since there was airway between the outer tube and inner tube, it was possible to continuously discharge the intra-abdominal fluid without damaging the tissue due to negative pressure.

**Figure 7:**
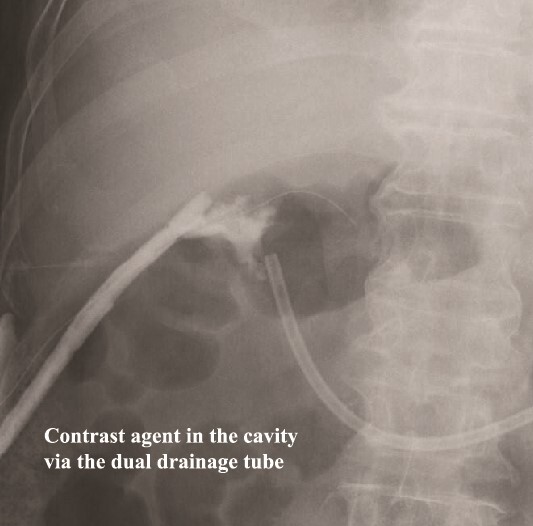
Fistulography locates the cavity, and the fistula is closed. As there was no flow of the contrast agent into the duodenum, we located the cavity around the duodenal stump.

**Figure 8:**
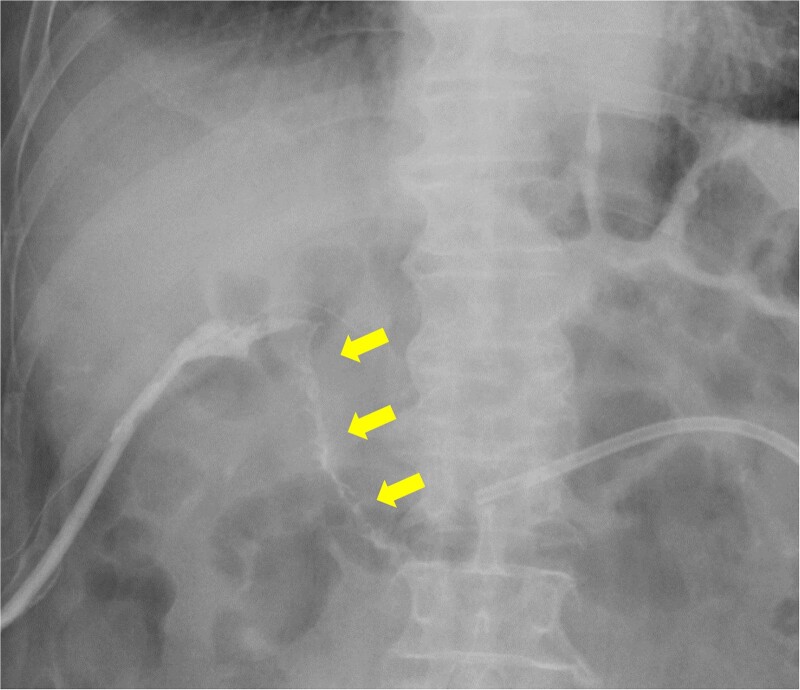
Fistulography reveals recurrence of the duodenal fistula. Fistulography at POD 44 showed recurrence of the duodenal fistula (arrows).

The amylase levels of drainage fluid and blood at postoperative day (POD) 1 (Ascites 6980 IU/dl, Serum 1123 IU/dl) and POD 3 (Ascites 673 IU/dl, Serum 487 IU/dl) revealed pancreatic fistula based on the criteria of International Study Group of Pancreatic Fistula [2]. After that, their results improved, and fluoroscopy showed no stenosis and leakage. Therefore, we removed all drains at POD 8, however the following day, the patient developed high fever. Plain CT revealed intraperitoneal fluid around subdiaphragmatic and duodenal stump (Fig. 3). We inserted a pig-tail drainage tube to the subdiaphragmatic space (Fig. 4). Since biliary fluid was discharged through the tube, we suspected duodenal leakage and started infusion of somatostatin analogs and antibiotics. At POD 13, the patient complained of whole abdominal pain with peritoneal signs. As the patient became hemodynamically unstable, we performed emergent laparotomy to lavage and insert multiple drainage tubes. Two perforation pinholes were identified in the anterior wall of the duodenum, near the stump. We resected the vulnerable duodenal stump including the perforation site and closed by Gambee’s method with unabsorbable 4–0 proline (Fig. 5). We inserted multiple drainage tubes (Fig. 6a): a C-tube from the cystic duct into the common bile duct to separate biliary juice and pancreatic juice, a dual drainage tube around the duodenal stump with continuous suction (Fig. 6b), a simple intraluminal drainage tube via the duodenum near the stump through a new skin incision on the left side of the abdomen for duodenal decompression, and a drainage tube into the rectovesical pouch. After the reoperation, we irrigated the cavity around the duodenal stump through each drain with saline. Since the contrast agent did not flow into the duodenum and the cavity around the duodenal stump gradually got smaller (Fig. 7), oral intake of fluid diet was initiated at POD 37. However, fistulography at POD 44 showed that the fistula of duodenal stump had relapsed (Fig. 8). Although we considered performing second reoperation for duodenal stump closure, due to the cavity around the duodenal stump was located, we continued conservative management and irrigation via drainage tubes. Fistulography demonstrated no leakage from the duodenal stump at POD 56, and the patient discharged at POD 59.

## Discussion

DSF is a dangerous complication of surgery for gastric cancer. It mainly occurs after Billroth-II or Roux-en-Y reconstruction. Its incidence rate is 1.5–2.5% of gastrostomies for gastric cancer and mortality rate is 12–28% [3–6]. Four management strategies for DSF have been reported: conservative, endoscopic, percutaneous, and surgical [7, 8].

In our case, DSF was likely caused by pancreatic fistula following lymph node dissection around the pancreas. However, duodenal thermal injury, or increased intraluminal pressure cannot be ruled out as causes. DSF may be caused by inadequate closure of duodenal stump or drain placement, inflammation and so on, but in most cases, the cause is unclear [9].

In our case, the peritoneal pig-tail catheter was not effective. Since the patient developed diffuse peritonitis and sepsis as a result, surgical lavage was necessary. Various surgical strategies for DSF, including lavage and drainage, have been reported [10–12]. It was reported that duodenostomy is preferred as a surgical treatment for DSF in acute phase to other methods because of its simplicity, minimal invasion, and flexibility [3]. According to a systematic review, the DSF-related mortality rate is 18.7%, and the success rate of surgical management for DSF is ~71.5% [8]. This high mortality rate is due to patients developing severe conditions such as septic shock or diffuse peritonitis.

In our case, we performed relaparotomy to diligently debride, in addition, insertion of the continuous suction dual drainage system around the duodenal stump to completely drain the digestive fluid, including pancreatic juice. Literature with modifications made directly to the drainage tube itself, as in the current case, is limited. However, a literature reported improvement in cure rates of DSF and postoperative hospital stay, among other factors, by catheter with an air vent compared to traditional closed drains [13]. The closed drains may have the risk of inducing peritonitis again in the event of drainage failure. Since there is airway between the outer tube and inner tube, it is possible to continuously discharge the intra-abdominal fluid without damaging the tissue due to negative pressure. In addition, we inserted the biliary drain with C-tube and intraluminal drain via the duodenum near the stump for biliary diversion, duodenal decompression, and further drainage of digestive fluid. Our case improved gradually without relapse of diffuse peritonitis and sepsis.

In our case, DSF recurred despite of the elaborate closure of the duodenal stump with unabsorbable proline by Gambee’s method. It was reported that because of inflammation and edema, surgical suture for closure of fistulas between 10 days and 6 weeks after initial gastric surgery was unfavorable [14]. Moreover, it was reported that purse-string suture is better than other methods to re-close the duodenal stump with DSF [15]. In our case, although DSF recurred, the multiple drainage tubes control it.

## Conclusion

DSF after gastrectomy for gastric cancer has risk of complications and high mortality. If the patient develops sepsis or diffuse peritonitis, relaparotomy may be required to rescue the patient. In this report, the multiple drainage system may be effective for surgical management for persistent DSF.

## References

[1] Sano T , Coit DG, Kim HH, et al. Proposal of a new stage grouping of gastric cancer for TNM classification: International Gastric Cancer Association staging project. Gastric Cancer 2017;20:217–25. 10.1007/s10120-016-0601-9. 26897166 PMC4992472

[2] Bassi C , Marchegiani G, Dervenis C, et al. The 2016 update of the International Study Group (ISGPS) definition and grading of postoperative pancreatic fistula: 11 years after. Surgery 2017;161:584–91. 10.1016/j.surg.2016.11.014. 28040257

[3] Ali BI , Park CH, Song KY. Outcomes of non-operative treatment for duodenal stump leakage after gastrectomy in patients with gastric cancer. J Gastric Cancer 2016;16:28–33. 10.5230/jgc.2016.16.1.28. 27104024 PMC4834618

[4] Paik HJ , Lee SH, Choi CI, et al. Duodenal stump fistula after gastrectomy for gastric cancer: risk factors, prevention, and management. Ann Surg Treat Res 2016;90:157–63. 10.4174/astr.2016.90.3.157. 26942159 PMC4773460

[5] Cozzaglio L , Giovenzana M, Biffi R, et al. Surgical management of duodenal stump fistula after elective gastrectomy for malignancy: an Italian retrospective multicenter study. Gastric Cancer 2016;19:273–9. 10.1007/s10120-014-0445-0. 25491774

[6] Aurello P , Sirimarco D, Magistri P, et al. Management of duodenal stump fistula after gastrectomy for gastric cancer: systematic review. World J Gastroenterol 2015;21:7571–6. 10.3748/wjg.v21.i24.7571. 26140005 PMC4481454

[7] Patricia YPC , Kevin WKF, Yee LF, et al. Duodenal stump leakage. Lessons to learn from a large-scale 15-year cohort study. Am J Surg 2020;220:976–81. 10.1016/j.amjsurg.2020.02.042. 32171473

[8] Zizzo M , Ugoletti L, Manzini L, et al. Management of duodenal stump fistula after gastrectomy for malignant disease: a systematic review of the literature. BMSC Surg 2019;19:55. 10.1186/s12893-019-0520-x. PMC6540539 31138190

[9] Ramos MFKP , Pereira MA, Barchi LC, et al. Duodenal fistula: the most lethal surgical complication in a case series of radical gastrectomy. Int J Surg 2018;53:366–70. 10.1016/j.ijsu.2018.03.082. 29653246

[10] Blouhos K , Boulas KA, Konstantinidou A, et al. Early rupture of an ultralow duodenal stump after extended surgery for gastric cancer with duodenal invasion managed by tube duodenostomy and cholangiostomy. Case Rep Surg 2013;2013:430295, 1–5. 10.1155/2013/430295. 24159410 PMC3789440

[11] Kamada Y , Hori T, Yamamoto H, et al. Treatment of labial fistula communicating with the duodenal stump after gastrectomy. Am J Case Rep 2019;20:851–8. 10.12659/AJCR.915947. 31203309 PMC6590267

[12] Furihata T , Furihata M, Satoh N, et al. Repeated duodenal stump perforation using a stapling device following subtotal gastrectomy with Roux-en-Y reconstruction for advanced gastric cancer: lessons from a rare case. Int Surg 2015;100:726–32. 10.9738/INTSURG-D-14-00266.1. 25875557 PMC4400946

[13] Wang X-T , Ya H-Q, Wang L, et al. Trocar puncture with modified sump drainage for duodenal stump fistula after radical gastrectomy for gastric cancer: a retrospective controlled study. Surg Open Sci 2023;16:121–6. 10.1016/j.sopen.2023.09.015. 37876666 PMC10590734

[14] Cozzaglio L , Coladonato M, Biffi R, et al. Duodenal fistula after elective gastrectomy for malignant disease: an Italian retrospective multicenter study. J Gastrointest Surg 2010;14:805–11. 10.1007/s11605-010-1166-2. 20143272

[15] Shao QS , Wang YX, Ye ZY, et al. Application of purse-string suture for management of duodenal stump in radical gastrectomy. Chin Med J (Engl) 2011;124:1018–21. 21542961

